# The Impact of Oxidative Stress of Environmental Origin on the Onset of Placental Diseases

**DOI:** 10.3390/antiox11010106

**Published:** 2022-01-01

**Authors:** Camino San Martin Ruano, Francisco Miralles, Céline Méhats, Daniel Vaiman

**Affiliations:** Institut Cochin Departement Development Reproduction Cancer, U1016 INSERM, UMR8104 CNRS, Université de Paris, 24 Rue du Faubourg St Jacques, 75014 Paris, France; camino.ruano@inserm.fr (C.S.M.R.); francisco.miralles@inserm.fr (F.M.); celine.mehats@inserm.fr (C.M.)

**Keywords:** environmental pollution, placenta, heavy metals, P38MAPK, DNA methylation, placental diseases, hypertensive disorders of pregnancy

## Abstract

Oxidative stress (OS) plays a pivotal role in placental development; however, abnormal loads in oxidative stress molecules may overwhelm the placental defense mechanisms and cause pathological situations. The environment in which the mother evolves triggers an exposure of the placental tissue to chemical, physical, and biological agents of OS, with potential pathological consequences. Here we shortly review the physiological and developmental functions of OS in the placenta, and present a series of environmental pollutants inducing placental oxidative stress, for which some insights regarding the underlying mechanisms have been proposed, leading to a recapitulation of the noxious effects of OS of environmental origin upon the human placenta.

## 1. Introduction

The placenta is an extraembryonic annex that develops as a major interface between the mother and the fetus in mammals. The placenta allows the exchanges of nutrients, gases, the discard of metabolic waste, and is operative for the synthesis of gestation-essential hormones responsible for placental growth, angiogenesis. The placenta is also essential for the immune tolerance of the mother towards the hemi-allogenic fetus and the dampening of the maternal immunological reactions throughout pregnancy.

One accompanying issue of placentation is the ubiquity of oxidative stress (OS). In a broad sense, OS is directly linked to the generation of chemicals that have a strong chemical affinity for biomolecules (proteins, lipids, DNA). When an excess of these reactive molecules is generated, it gives rise to OS. Despite being necessary for proper placental development, OS can have deleterious effects if an extreme dysregulation occurs. Elevated OS can induce complications in pregnancy progression, leading to long-term effects on both the mother and the fetus. These links were recently reviewed for recurrent pregnancy loss (RPL) [1], prematurity [2], intra-uterine growth restriction (IUGR) [3], diabetes [4], and preeclampsia, PE [5].

## 2. Reminders about Hypoxia, Oxidative Stress, and Placental Development

**Figure 1:**
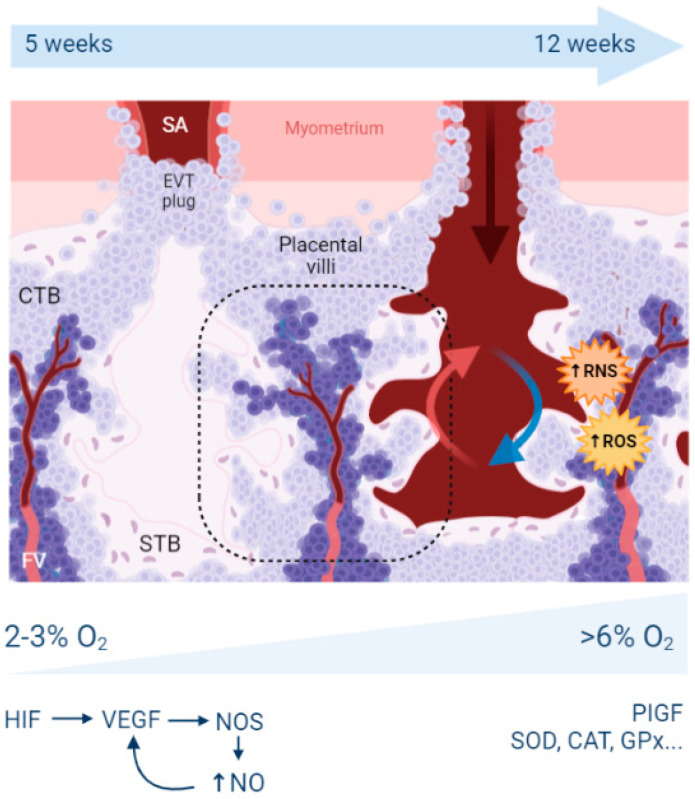
Schematic representation of a placental villous tree during human pregnancy. These tree-like structures are composed of an outermost syncytiotrophoblast (STB) layer in pink, cytotrophoblast (light purple) and fetal stromal cells (dark purple). Fetal capillaries invade these structures to obtain nutrients and exchange gases. Between each placental villous, maternal lacunae are visible, where the maternal spiral arteries potentially deliver the maternal blood. In the early stages of pregnancy, this supply is blocked by extravillous trophoblast plugs, leading to a low oxygen concentration (2–3% O_2_). Expression of hypoxia-inducible transcription factors (HIF) stimulates the expression of the vascular endothelial growth factor (VEGF), which stimulates the production of nitric oxide (NO). NO stimulates, at the same time, VEGF functions as a pro-angiogenic molecule. Once these plugs are removed around week 12 of pregnancy, the maternal blood bathes the lacunae, allowing the interchange of nutrients and gases between the mother and the foetus. This highly oxygenated blood increases the oxygen tension to normoxic levels (6–8% O_2_ in the tissue context), increasing the STB mitochondrial activity and thus, the release of reactive oxygen species (ROS) and reactive nitrogen species (RNS). Increased OS induces the expression of antioxidants such as superoxide dismutase (SOD), catalase (CAT), glutathione peroxidase (GPx). Oxygen tension stimulates the placental inducible growth factor (PIGF) implicated expression in endothelial growth and angiogenesis.

Morphologically, the placenta comprises two specific cell types: the cytotrophoblast (CTB) and the syncytiotrophoblast (STB). Other cell types are also found in other tissues, such as endothelial cells, mesenchymal cells, and macrophages (named Hoffbauer cells in the placenta). CTB are mononucleated cells that can fuse and differentiate into STB, a syncytium where nuclei share the same cytoplasm. Both cell types are organized in highly branched (up to 16 degrees of ramification) tree-like structures called placental villi, which are embedded in spaces filled by maternal blood, constituting the intervillous space or lacunae (Figure 1). Inside the villi and their neighboring surface, fetal capillaries are located and collect the nutrients and gases which have passed or diffused through the different trophoblast layers.

The insertion of the embryo inside the maternal uterus is a multi-step process. From very early stages (as soon as the fusion occur, immediately after the blastocyst implants), STB secretes matrix metalloproteases that will facilitate the implantation. In parallel, extravillous trophoblast (EVTs) will prepare later stages of the invasion process (after the second trimester of gestation in women), when angiogenesis becomes crucial to promote fetal growth. Part of these EVT will mimic endothelial cells to substitute for the maternal endothelial cells of the maternal uterine spiral arteries. These EVTs disrupt the vascular smooth muscle layer and generate a different, non-contractile vascular function. The arteries switch from narrow, muscular, and low perfusion vessels into dilated sinuses without vasomotor function. A proper remodeling, in which trophoblast cells substitute the maternal endothelial cells until the inner myometrial zone, also called the junctional zone [6], is necessary to supply the growing demands of the fetus from the second trimester of pregnancy.

During the first weeks of pregnancy, the embryo is in a low oxygen concentration environment. The trophoblast cells form EVTs plugs in the uterine arteries, blocking maternal blood circulation into the lacunae and thus thwarting oxygenation. This hypoxic state is crucial for placenta development, expediating trophoblast invasion into the maternal decidua. Trophoblast cellular functions and transcription is regulated by the two main Hypoxia-Inducible Factors (HIF-1 and HIF-2). These two transcription factors bind to specific DNA sequences called hypoxia response elements (HREs), present in more than 200 genes. HIF-1 is a heterodimeric protein composed of an oxygen-sensitive subunit (HIF1-α) and an oxygen-independent subunit (HIF-1β) [7]. In a hypoxic environment, HIF1 is stabilized, functional, and will, in particular, stimulate the expression of the vascular endothelial growth factor (VEGF), a key player in vascularization, branching, angiogenesis, and endothelial proliferation [8]. VEGF depends on nitric oxide (NO), which is synthetized by NO synthases on STB and fetal endothelial cells (in particular, iNOS—the inducible gene and eNOS—the endothelial-specific gene). NO stimulates angiogenesis, vessel formation, vascular tree maturation, and organization. In the specific case of PE, measures along the three trimesters were performed in the placenta for SOD, GPx, and eNOS enzymes [9]. GPx was significantly higher during T3 (third trimester of pregnancy); SOD was lower during T2 (second trimester of pregnancy) and T3. GSH levels and GPx were lower in PE than in controls at T1 (first trimester of pregnancy).

Once the EVTs plugs are expelled, oxygenated blood enters the placenta and stimulates the second wave of trophoblast invasion, expanding further the maternal arteries. This increase in oxygen tension induces an increase of OS, which extends from the periphery to the center of the placenta. Normoxia stimulates Placental Inducible Growth Factor (PIGF) expression, which binds to VEGFR-1 (Vascular Endothelial Growth Factor-1 Receptor-1) or FLT-1 (Fms-related tyrosine kinase-1), stimulating endothelial growth and non-branching angiogenesis [10]. In parallel, mitochondrial activity is upregulated, triggering OS by the production of reactive oxygen species (ROS) and reactive nitrogen species (RNS) [11]. These RONS (ROS and RNS) species react with NO, decreasing the bioavailability of this molecule and producing harmful molecules with cell detrimental upshots such as peroxynitrite (ONOO-). The placenta, to counterbalance this burst of OS, upregulates the expression of antioxidants such as superoxide dismutase (SOD), catalase (CAT), glutathione peroxidase (GPx), etc. [12]. Nevertheless, the increased concentrations of RONS may overwhelm the defense systems and eventually induce the expression of inflammatory cytokines (IL-1α, IL-1β, IL-6, IL-8, TNFα, CCL2) and the augmentation of arterial stiffness. This supports the hypothesis that in pathological placentas, such as in PE and IUGR, extremely high concentrations of OS result in endothelial dysfunction, a condition triggering systemic inflammation, vasoconstriction, and prothrombotic propensities.

### 2.1. What Is the Origin of Oxidative Stress in the Placenta?

As in other tissues, OS in the placenta can originate from two main sources: an increase in the RONS concentration or a decrease in the total antioxidant capacity.

RONS production in the placenta is highly restricted to the mitochondria in both physiological and pathological conditions. Mitochondria suffer from electron leakage during the oxidative respiration step, where 1–2% of the oxygen is converted into the superoxide radicals, such as O_2_^•−^. SOD activity reduces these radicals into H_2_O_2_, a molecule that can move to the cytosol, and that will be further reduced into water by GPx [13]. Mitochondria found in the two major placental cell types (STB and CTB) are morphologically unique and show different bioenergetic properties. CTB cells have stereotypical mitochondria, of 0.2–0.8 μm, which change after differentiation into STB. Mitochondria located in the syncytium are spherical and smaller, with structural changes triggering decreased respiratory capacity, decreased ATP production rate and enhanced electron leakage [14]. Moreover, STB mitochondria are highly implicated in steroidogenesis, and progesterone biosynthesis needs a high level of energy generated by the mitochondria. Indeed, the protein StAR located at the outer mitochondrial membrane interacts with TSPO, allowing the cholesterol to translocate inside the mitochondria, to be changed into pregnenolone, which will later be transformed into progesterone via the 3β-HSD pathway [15]. In addition to enhanced RONS production and higher O_2_ concentration changes, STB cells do not synthesize enough levels of SOD; thus, reduction of the ROS species cannot occur, increasing the sensitivity of the syncytium to OS and leading to high levels of lipid peroxidation, cell damage, and senescence. This senescence rate is exacerbated in the context of pathological pregnancies; for example, in PE, STB stress signals in maternal circulation are used as biomarkers for the pathology [16].

To note, mitochondria from female fetuses produce less ROS than male mitochondria. This can be linked with the increased concentrations of antioxidants in females due to the action of estrogens. This leads to differential ways of improvement being at work in male and female fetuses following anti-oxidant treatments [17].

### 2.2. What Are the Antioxidant Mechanisms in the Placenta?

As mentioned before, under OS, the placenta expresses different antioxidants to counterbalance the production of RONS. Inadequate concentrations of antioxidants can lead to an insufficient RONS elimination, resulting in increased OS. There are two different types of antioxidants: enzymatic (CAT, SOD, GPx, glutathione reductase, thioredoxin oxidase…) and non-enzymatic (vitamin C and vitamin E, Selenium). The details are presented in [12] to which the reader can refer to have a description of these mechanisms; in short, SOD is able to transform the hypertoxic superoxide ions O_2_^•−^ (often from the mitochondrial respiratory chain) into hydrogen peroxide, H_2_O_2_, to be degraded into the innocuous molecules O_2_ and H_2_O, through two pathways, the catalase pathway and the glutathione pathway. A recent study performed a dosage of the key antioxidant proteins in human placentas from 50 controls and 100 preeclamptic women, revealing excess of catalase, SOD, GSH/GSSG ratio, IL6, and lower GSSG in the PE women, suggesting that in PE-derived placentas an adaptor mechanism triggers a big part of the antioxidant machinery [18].

Catalase is a heme-containing oxidoreductase composed of four 527 amino-acid polypeptides encoded by a gene located in 11p13 in humans. The iron atom in the heme is able to catalyze the lysis of the covalent bond linking the two oxygen atoms of hydrogen peroxide. There are links between the various antioxidant pathways at work in the placenta, since it has recently been shown that selenium depletion in the placenta is associated with decreased CAT and GSH concentrations and increased H_2_O_2_ [19].

Lower expression of glutathione reductase is observed in preeclamptic placentas, creating higher ratios of oxidized:reduced glutathione [20]. On the other hand, increased expression of thioredoxin peroxidase-3 (TPx) was observed to be increased 3.25-fold compared with normal healthy placentas. However, there is no increased expression of its couple, thioredoxin reductase, which may limit the activity of TPx [20].

Besides the endogenous mechanisms devoted to the management of oxidative stress in the placenta, there are systemic actions that may have antioxidant potential. For instance, recent evidence suggests that maternal supplementation of magnesium could be a way of alleviating the effects of oxidative stress. In fact, in maternal erythrocytes, magnesium modulates the rhythm of exchange of ions (especially SO4^2−^) through the protein AE1 (aka BAND3), and this leads putatively to a stabilization of the membrane structure, increasing their homeostasis and thus improving the resistance to oxidative stress-connected diseases [21,22], such as preeclampsia/eclampsia, diseases well known to be sensitive to magnesium supplementation [23].

Besides internal (meaning genetic, developmental, etc.) reasons leading to abnormal exposure to oxidative stress, environmental sources can provide pro-oxidant molecules or resorb the antioxidant capabilities of the placenta.

## 3. Mechanisms of Oxidative Stress Induction by Environmental Pollutants

Environmental pollutants are produced mainly as a consequence of human activities, but also by other living organisms or natural causes, and are ubiquitously distributed in air, soil, water, food, plastics, etc. Therefore, all living organisms, including humans, are exposed to a variety of pollutants throughout their life. Epidemiological evidence shows that many of these pollutants can adversely impact pregnancy. Pending on the nature of the pollutant, the health of the mother, the fetus, or both, can be seriously affected. The toxic effects of pollutants can lead to embryonic mortality, spontaneous abortion, IUGR, low birth weight, neurophysiological pathologies, etc. [24,25,26,27]. The mechanisms of action of these pollutants are diverse, but in many cases, induction of OS has been identified as a common mechanism of action [26,28,29]. In this section, we will thus focus on those environmental pollutants which have been found in the placenta or conceptus and are known to act through the induction of OS. These pollutants belong to several categories, heavy metals, air pollutants (fine particles, tobacco smoke), pollutants of biological origin (toxins), industrial molecules used in the production of plastics, and domestic goods (phenols and parabens). Here we will focus on the cases where some degree of mechanistic information has been gained through research.

The enhancement of ROS production and the induction of cellular damage via OS is a common finding in the studies analyzing the mode of action of environmental pollutants. In most cases, however, the precise mechanisms underlying the increased production of ROS by environmental pollutants remain to be discovered and analyzed. Nevertheless, several studies distinguish between direct and indirect mechanisms. Direct mechanisms involve the production of ROS as a result of the chemical reaction of the pollutant with other molecules present inside the cell. Indirect effects involve the capacity of some pollutants to inhibit ROS scavenging antioxidant enzymes, but also their capacity to inhibit the electron transport chain (ETC) of the mitochondria with the subsequent increase in superoxide production and decreased ATP generation.

### 3.1. Exposure to Environmental Metals

The induction of ROS production has been studied with some detail in the case of heavy metals [30]. These exposures are detectable in the maternal blood. This has recently been studied for 41 metals and metalloids in the Shanxi province of China [31]. OS is detectable on DNA through 8-OHdG labeling; 8-Oxo-7,8-dihydroguanine (8-OHdG) is widely used as a predominant biomarker for OS, with the advantage of being relatively stable and easy to study by validated ELISA tests [32]. The Singh study attempted to connect the metal/metalloid exposure to OS to the risk of spontaneous preterm birth (74 cases, 74 controls). The study revealed that exposure to lutetium, erbium, europium, praseodymium, and iron indeed increased the marks of OS in the maternal DNA. This suggests that placental alterations similar to those of the maternal DNA are plausible, although they have not been directly studied by the authors.

#### 3.1.1. Cadmium

A series of relevant papers present the cadmium/selenium metal balance as a key in the management of OS in the placenta.

Cadmium (Cd) is found in the earth’s crust in combination with oxygen, chlorine or sulfur, and was extensively delivered in the outside through anthropic industrial activity [33]. As nicely reviewed in 2010, multiple studies demonstrated its implication in oxidative stress in various organs and tissues [34]. Cd-induced OS results from the mitochondrial ETC inhibition, the displacement of redox-active metals, the depletion of antioxidants, the inactivation of antioxidant enzymes (through interference with -SH groups and replacement of necessary cofactors) and the activation of NADPH oxidases [35,36]. The mechanism of ROS induction by Cd has been recently reviewed [37]. The Fe-S clusters of the ETC complexes have been identified as Cd targets sites, resulting in electron transfer inhibition and the dissipation of proton electrochemical gradient necessary for ATP synthesis. Cd has also been shown to induce ROS generation by mitochondrial permeability transition pore (MPTP) opening, leading to cytochrome c release. Since cytochrome c is responsible for the electron transfer from Complex III to Complex IV, the release of cytochrome c disrupts the mitochondrial ETC, causing further ROS. Moreover, Cd induces OS by intracellular GSH depletion.

The cytotoxicity of Cd has been addressed in trophoblast cell models [38], where exposure altered the cell organelle structure, decreased cell viability, and increased expression of oxidative stress-managing genes (SOD1, ROS1, HSPA6). The dosage of Cd can be performed from easily accessible tissue such as toenails [39], which allows us to evaluate putative effects on pregnancy outcome. In this recent study, Cd tended to be associated with a trend towards unfavorable pregnancy outcomes. Synergistic effects also exist with other metals (such as Arsenic, As), as shown in [40] in the JEG-3 trophoblast cell model. In this simplified model, the exposure to Cd/As mixtures led to a synergistically increased expression of the mRNA from Heme oxygenase1, metallothioneins MT1A, MT1F, and MT1G, after 24 h of exposure. A recent in vitro study shows that Cd induces OS in HTR-8/SV-neo cells, another trophoblast cell model. Cd causes distortion of mitochondrial structure, reduction of mitochondrial membrane potential, DNA damage and G0/G1 phase arrest. In addition, Cd treatment increases Bax/Bcl-2 ratios and leads to HTR-8/SV-neo cell apoptosis.

Cd is also a major constituent of tobacco smoke [41,42] and could contribute to the numerous general effects connected to cigarette smoke-related OS, particularly in the placenta (similar to what is particularly well documented in lungs [43]). Maternal exposure to Cd has been associated with pregnancy complications, including early delivery and low birth weight. [44,45,46]. Experimental studies in rats exposed to Cd from GD 9 to 21 have shown increased H_2_O_2_ production and lipid peroxidation. This was correlated with a decrease in the activity of key ROS scavenging enzymes SOD, CAT, GPX, glutathione reductase, and glutathione-S-transferase [47]. N-acetyl-l-cysteine (NAC), a ROS scavenger, significantly attenuates Cd-caused mitochondrial injury [48]. In the specific case of IUGR, a dosage of antioxidant molecules (glutathione, SOD, Glutathione peroxidases) in a 2009 study did not associate OS parameters with low birth weight [49] under exposure to metals that were systematically measured in this last study in the placenta, by instrumental neutron activation analysis (INAA), i.e., Zn, Hg, Se and As, and by atomic absorption spectrometry (AAS), i.e., Fe, Cu, Cd. In the case of preterm birth, by contrast, a recent review [50], based upon the cross-analysis of 36 articles, showed that there is a higher incidence of the disease under exposure to Pb and Cd, while the results were inconclusive for Hg and As.

In terms of mechanisms, Cd exposure reduces the level of progesterone (a major tolerogenic pregnancy hormone) through the downregulation of enzymes involved in progesterone synthesis (StAR, CYP11A1). It also leads to mitochondrial stress (and therefore to OS) by activation of the GCN-2/p-eIF2α signaling pathway in the trophoblasts [51]. In connection with what was previously mentioned regarding the beneficial role of magnesium on oxidative stress, cadmium also has effects on exchange capabilities through modulating the activity of the AE1 (BAND3) protein [52].

#### 3.1.2. Mercury and Methyl-Mercury

Methylmercury (MeHg) is another heavy metal derivative known to enhance ROS production by inhibiting the ETC of mitochondria. Inhibition of the complexes III and IV of the mitochondrial respiratory chain was observed in rat primary cerebellar granule neurons treated with MeHg [53]. This was accompanied by increased mitochondrial-derived superoxide (O_2_^−^) production, decreased ATP production, the disruption of mitochondrial membrane potential, and the opening of MPTP. In addition, MeHg inhibits the activity of GPx by direct binding to its selenocysteine group. Inhibition of GPx results in higher ROS levels and lipid peroxidation in vitro in the neuroblast SH-SY5Y cells and mitochondria-enriched fractions from MeHg-exposed mice [54]. Finally, it has been observed that MeHg also decreases the protein expression of TrxR, greatly reducing the Trx activity in both the cytosol and mitochondria [55].

Human exposure to MeHg generally results from the consumption of contaminated fish and shellfish. Since MeHg easily crosses the placental barrier, it is particularly toxic to the developing fetus, resulting in complications such as spontaneous abortions, stillbirths, physical malformations, motor impairment, cognitive delay, and behavioral abnormalities [56]. MeHg accumulates in the placenta, and it has been proposed to be readily transported across the placenta bound to thiol-groups in cysteine, thereby mimicking methionine [57,58]. The formation of thiol-MeHg bonds is likely also responsible for the placental accumulation because MeHg, similar to Hg^2+^, is known to bind to thiol-containing molecules—for example, proteins, cysteine, and glutathione. Recently the amino acid transporter LAT1 (SLC7A5) has been involved in the transport of MeHg in HTR-8/SV-neo cells [59]. Regarding the actual mechanism of action of MeHg, it has been demonstrated that 1 μg/mL methylmercury decreased viability, proliferation, and migration and led to the decreased expression of SOD1 in the HTR8/SV-neo trophoblast cells [60].

#### 3.1.3. Lead

Lead (Pb) is a ubiquitous environmental pollutant with high neurotoxic potential. Pb, as well as Hg, are well-known to cross the placenta and accumulate in fetal tissues. Prenatal exposure to lead produces toxic effects in the human fetus, including an increased risk of preterm delivery, low birth weight, and impaired mental development. Pb causes oxidative damage to lipids and DNA [61,62,63,64], resulting in leaky membranes and apoptosis. In a study performed in rats exposed in utero to a low dose of Pb, levels and activities of Cu/Zn-SOD, Mn-SOD, GPx1, and GPx4 were decreased in the hippocampus [65]. Changes in mitochondrial antioxidant enzymes occur during in utero exposures to Pb and persist in rats past postnatal day 35 [66]. Inhibition of these enzymes is due to the ability of Pb to substitute for the divalent metals necessary for enzymatic function. In addition, GSH levels were diminished by Pb exposure [65]. OS can result in mtDNA (mitochondrial DNA) damage which mitochondria attempt to compensate this effect by increasing mtDNA content [67]. A recent study has shown that maternal second and third trimester, as well as cord blood Pb levels, are associated with an increase of cord blood mtDNA content and that preterm delivery situation marginally synergize with Pb to increase mtDNA content in cord blood [68].

#### 3.1.4. Chromium

Hexavalent chromium (CrVI) is used in numerous industries, including leather, textile, metallurgical, chemical, and automobile [69]. Due to wide use and improper disposal, it contaminates water, soil, and air. Epidemiological studies examining women working in industries using CrVI found high levels of Cr in the blood, urine, umbilical cord, and placenta. A clear association has been established between CrVI exposure and pregnancy complications, including preterm labor, spontaneous abortion, and IUGR [70,71,72]. Exposure of pregnant rats to CrVI through drinking water results in Cr accumulation in the placenta [73]. CrVI passes into the cells by anionic transporters and is converted into CrIII by antioxidants such as ascorbic, acid, glutathione, N-actyl-cysteine, and enzymes such GPX, SOD, and Catalase [74]. This reduction process generates enormous amounts of ROS, which increase oxidative stress. Banu and coworkers have shown that the oral administration of CrVI, at environmental doses (50 ppm) to pregnant rats from GD 9.5–14.5 results in IUGR [75]. Examination of the placentas at GD 18.5 shows that CrVI exposure disrupts trophoblast proliferation, increases ROS and decreases the expression of antioxidant enzymes GPX1, SOD1, SOD2, PRDX3, and TXN2.

### 3.2. Exposure to Tobacco

Tobacco exposure of the placenta concerns directly 5 to 20% of women that smoke during pregnancy in the USA and in Europe. Smoking during pregnancy is known to contribute to numerous poor birth outcomes, such as low birth weight, preterm birth as well as life-long health and developmental problems [76]. One recent observation is that this exposure affects epigenetic marks, and in particular, DNA methylation sometimes irreversibly, even if the women stop smoking pre-pregnancy [77]. These methylation alterations (203 significant Differentially Methylated Regions) are not distributed randomly but co-localize with specific ablation of chromatin marks, such as the trimethylation of histone H3 (H3K4me3), and gain of other marks such as H3 methylation at the lysine 27 (H3K27ac). Amongst the 203 DMR, one found MAPK8 embedded in a network of genes involved in stress sensing (including oxidative stress). This gene network is in particular involved in the context of myocardial remodeling [78]. The effects of tobacco are also visible in terms of placental morphology [79], inflammation mechanisms via prostaglandin metabolism [80]. The observed effects could be mediated by exosomes [81]. Exosomes isolated from the BeWo trophoblast cell model exposed to tobacco smoke induce inflammatory changes in uterine cells [82]. The link of tobacco exposure with OS was unsuccessfully explored through the expression of candidate genes [83], while specific markers of oxidative stress are indeed altered, such as malondialdehyde (MDA) or the total antioxidant capacity (TAC) in the amniotic fluid [84]. Other markers of cellular stress were also altered in the fetal membranes, such as F2-IsoProstane, together with a decrease of Bcl2 expression, and increase of caspase 3.

**Figure 2:**
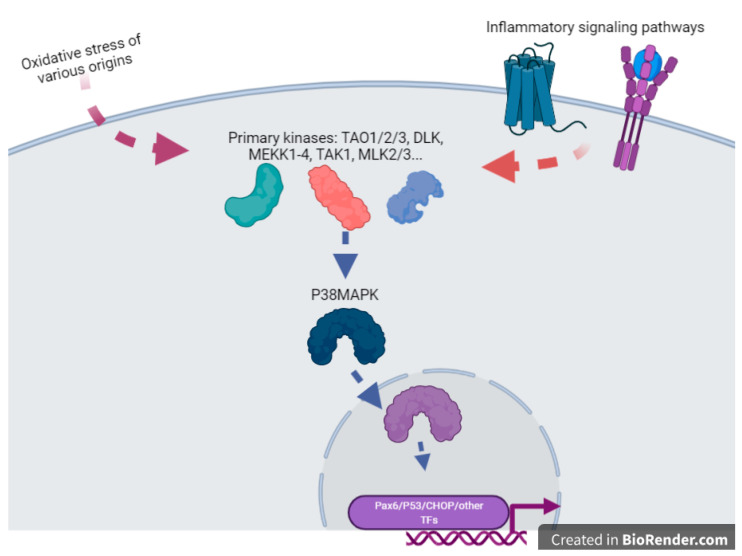
A cartoon emphasizing the pivotal role of P38MAPK(MAPK11) in the sensing of OS and the direct or indirect activation of various transcription factors. This is an oversimplification since this factor, once inside the nucleus, can also modulate chromatin structure through action upon structural components of the chromatins such as HMGN1 or Histone H3. This drawing is a simplification from [89].

Cigarette smoke extract exposure in vivo by injection in the uterine sacs of mice induce specific protein damages, protein nitrotyrosylation [85], especially with the activation of p38MAPK (aka MAPK11), a major actor of inflammatory processes, such as TLR signaling [86]. In these reactions, the activity of p38MAPK seems crucial (Figure 2), and the activity of this enzyme appeared induced by OS leading to inflammation in various cell types, and various studies indicate that this molecule can be relatively specifically targeted to alleviate defects induced by OS, for instance with specific inhibitors (SB203580) or statins, molecules that are well-known for their anti-cholesterol effects. P38MAPK could be important for the regulation of senescence, and all the senescence processes are pivotal in gestation since they are the monitors of placental ageing. Abnormal regulation of placental ageing has been involved in various placental pathologies, such as PE or the premature rupture of fetal membranes, leading to preterm birth. Tobacco smoke extracts accelerate the senescence, and there are concrete elements linking in a cascade: tobacco smoke ➔ oxidative stress ➔ p38MAPK activation ➔ senescence ➔ placental disease; these mechanisms may be counterbalanced by simvastatin and rosuvastatin treatment [87]. This implication of the p38MAPK cascade was also evinced in the mouse model [85]. Three kinase signaling pathways co-exist in eukaryotic cells (p38MAPK, ERK, and JNK (aka MAPK8)). The two first kinases can activate the COX-2 (PTGS2) prostaglandin inflammatory pathway (leading to PGE_2_ synthesis), while the JNK pathway tends to allow direct biogenesis of PGE_2_ without activating PTGS2. Similar to p38, cigarette smoke extract may also act through ERK1/2 activation [88]. This suggests that activation of ROS through the ERK1/2-EGR1 axis may also potentially contribute to the production of PlGF since EGR1 binds to the PlGF promoter. In this case, since PlGF is a favorable cofactor in pregnancy, this pathway could represent a normal contribution of ROS to increased angiogenesis in the early placenta, after the spiral arteries trophoblast plugs are expelled, at the dawn of the second trimester of pregnancy.

Exposure to smokeless tobacco has recently been shown to induce OS (through the analysis of DNA damage) in the placenta in regions where the active principles are consumed without smoking [90]. In this case, hypoxic markers (HIF1α) are increased together with the 8-OHdG OS mark on the placental cell DNA molecules. This was indirectly associated with low birth weight in which placental vasculogenesis appeared reduced compared to normal birth weight placentas. Some studies argued that as a principal component of tobacco smoke, nicotine alone is responsible for the majority of adverse reproductive effects. However, tobacco smoke is a toxic mixture of more than 5000 chemicals. In addition to nicotine, tobacco smoke contains toxic heavy metals (Cd, hexavalent chromium, and nickel), polycyclic aromatic hydrocarbons (PAH), nitrosamines, phenol, toluene, formaldehyde, etc. [42]. Active or passive maternal tobacco smoking is associated with important alterations in oxidant and antioxidant balance in fetal cord blood and causes potent OS [91]. It has also been evoked an influence of tobacco-induced OS susceptible to affect the normal function of fetal membranes at delivery time [92]. By an in vitro approach mimicking the stretching of fetal membranes using Amnion Epithelial Cells submitted to stretching, there was an enhanced activation of p38MAPK, an increase of senescence markers and an increased level of MMP9. These effects were associated with the production of exosomes by the AEC, where the phosphorylated form of p38MAPK is more abundant and contain an altered cargo of 221 different proteins compared to non-exposed patients [81]. In addition, a strong association has been reported between maternal tobacco use and aberrant placental metabolism, syncytial knot formation, and increased oxidative damage [93]. The same study also reported an increased expression of *CYP1A1*. This enzyme metabolically activates PAH compounds into oxidized derivatives, resulting in reactive oxygen intermediates capable of covalently binding DNA to form adducts [94]. One of the observed effects of tobacco is the decreased levels of genes encoding glutathione-S transferases (GSTM1, GSTA), which contribute to the GSH conjugation and GSH-dependent biotransformation of xenobiotics [95]. In pregnant rats exposed to cigarette smoke for 21 days, the activities of SOD and GPx were markedly decreased in the placenta. Exposition to cigarette smoke diminished the antioxidant capacities in the pregnant animals [96].

A striking question regarding tobacco smoke exposure and specifically PE and gestational hypertension resides in an observation that is not shared with other diseases of placental origin (low birth weight, spontaneous abortion, or risks of ectopic pregnancies); this observation is that surprisingly, but consistently in the literature, smoking women appear protected against PE. As an example, a recent paper analyzing the 2015 US natality records indicated that women that smoke before and during pregnancy had a reduced risk of gestational hypertension (RR = 0.92, 95% CI 0.90–0.94); contrastingly, women that quit smoking before pregnancy were at increased risk (RR = 1.02, 95% CI 1.00–1.05), and this was similar when quitting occurred up to the third trimester of pregnancy [97]. This recent observation suggests a vulnerability window, which was also observed recently in the risk of preterm birth, where the hazard comes more from changing the smoking habit than to the absence of modification of it [98]. The origin of this mild protection offered by smoking is still debated, with some consistent reports pointing at a direct specific function of nicotine, modulating the action of the nicotinic receptors in the placenta [99]. In the rat RUPP surgical model of preeclampsia, nicotine was indeed able to reduce placental ischemia, without specific effects on the component of the complement function [100]. Some observations also suggest that nicotine enhance the secretion of VEGF by trophoblast cells in hypoxia, presumably through the HIF1α sensing of oxygen shortage, this enhancing the physiological function of endothelial cells [101]. Other important antioxidant pathways, such as the heme oxygenase enzyme (HO-1) and its metabolite, carbon monoxide (CO), which can reduce the production of sFlt1 and sEng, could be an explanation of the relative protection of cigarette smoke, although no direct evidence is currently available [102]. The idea of CO being operational in the protection was evoked in detail in 2005 [103]. In this hypothesis paper, it is proposed that CO increases trophoblast invasion and arteriole remodeling, decreases apoptosis of the SCT, increases the materno–fetal blood flow, activate hemoproteins improving endothelial function, and activatee the antioxidant systems of the placenta. Another study on the rat model showed that a donor of H_2_S (a gas known to have vasodilatory effects), produced from the degradation of proteins and through the action of the CSE (Cystathionin γ-lyase protein) induced a decrease of placental oxidative damage (8-OHdG, MDA) induced by exposure to cigarette smoke. In addition, the GSH/GSSG ratio increased while antioxidants (SOD1, SOD2, CAT, GPx activities and expression) were increased; it is proposed that the key factor Nrf2 (decreased by CSE) is reestablished to normal levels [104].

### 3.3. Exposure to Airborne Particulate Matter

Airborne particulate matter (PM) is an important pollutant of the urban atmosphere and has been linked to OS and inflammation [105]. The particles are classified according to their diameter as PM2.5 or PM10 for example (<2.5 or 10 µM, respectively). It has been shown that air pollution particles may translocate through the STB membrane and cross the placental barrier [106]. PM from combustion sources contains a number of constituents that generate ROS by a variety of reactions [107]. A population study published in 2016 indicates clearly that this OS is combined with a proportion of nitrosative stress, a type of stress promoted by the generation of peroxynitrite molecules, ultimately leading to the covalent coupling of 3-nitrotyrosine to proteins causing their altered function [108]. This specific mark has recently been shown to be a premise of neurogenerative diseases, present before the onset of symptoms [109]. In the paper by Saenen and coworkers [108], 330 mother newborn couples revealed an increase by 35% of the level of nitrotyrosinated proteins, in association with an increase of 29% or 39% of the PM2.5, according to definite exposure windows (first and second trimester, respectively, while the third was not significant). In addition, it has now been shown that exposure to PM leads to locus-specific DNA methylation alterations [110]. One of the clearly identified loci was in the vicinity of the gene ADORA2B. Interestingly, this gene was associated to hyperglycemia and OS in several contexts, in particular gestational diabetes mellitus [111,112].

Several publications relate air pollution particle exposure to the oxidation of DNA [113]. In nuclear and mitochondrial DNA (mtDNA), the free radicals induced oxidative lesions. An examination of the effects of PM (2.5 and 10 μm diameter) exposure during pregnancy revealed an associated increase of mitochondrial 8-OHdG in maternal and cord blood of newborns. Thus, air pollution exposure in early life has a role in increasing systemic oxidative stress and DNA damage, at the level of the mitochondria, both in the mother and foetus [114]. Another study found an association between PM2.5 μm exposure during pregnancy and placental mtDNA methylation (especially in the MT-RNR1 region). According to the authors, this increased mtDNA methylation could reflect signs of mitophagy and mitochondrial death and result from PM induced oxidative stress [115].

### 3.4. Exposure to Vexing Biomolecules and Riling Toxins

Deoxynivalenol (DON) belongs to the type B group of the trichothecenes family, which is composed of sesquiterpenoid metabolites produced by Fusarium and other fungi in crops used for food and feed production [116]. Epidemiological studies have documented that DON affects animal and human health by causing various toxicities [117]. DON can transport across the placental barrier [118], and in pregnant mice, a relatively low-dose maternal DON exposure can result in developmental toxicities for embryos [119]. DON induces excessive accumulation of ROS, which leads to structural and functional damages of the placenta, causing adverse pregnancy outcomes. An experimental study using pregnant mice and BeWo cells has shown that DON exposure activates the Nrf2/HO-1 pathway and the expressions while its downstream antioxidant enzymes are increased to protect the placenta against oxidative stress [120,121]. However, with a longer time and higher dose exposure, the antioxidant capacity of cells reaches its limit. According to the authors, the reason of this “threshold effect” could be a consequence of the continuous release of iron by the HO-1 activity. This iron would then be involved in deleterious reactions that compete with iron reutilization, sequestration pathways, and aggravate oxidative stress [122].

The T-2 toxin is another major Fusarium mycotoxin contaminating crops [123]. In pregnant animals, the T-2 toxin induces placental lesions, embryotoxicity and abnormal development of offspring [123,124]. It has been shown that the T-2 toxin induces OS in placenta; however, its precise mode of action remains to be explored [125].

Microcystin-LR (MCLR) is another toxin able to impact human and animal reproduction [126]. MCLR is an environmental pollutant released by cyanobacteria in freshwater [127]. MCLR is actively absorbed by animals, fish, and birds from intoxicated water and thus enter the food chain. Humans are also exposed to microcystins, for instance, when they perform leisure activities in contaminated water. Pregnant mice intraperitoneally injected with MCLR (5 or 20 μg/kg) from gestational day (GD) 13 to GD17 present with reduced placenta and offspring weight. Expression of genes encoding placental growth factors Vegfα and Pgf, and transport pumps Glut1 and Pcft are in this case dampened in the placentas. Moreover, significant increases in MDA revealed the occurrence of OS caused by MCLR, which was also verified by a remarkable decrease in the glutathione levels, total antioxidant capacity (T-AOC), as well as the activity of antioxidant enzymes [128]. Moreover, MCLR activates the endoplasmic reticulum (ER) stress pathway in the placentas.

While maybe less understood at the molecular level, it is worth mentioning given its historical importance the case of dioxins for which a wide literature exists following the Seveso catastrophe, when in 1976, a chemical plant accidentally released a dioxin derivative, the TCDD (2, 3, 7, 8-tetrachlorodibenzo-p-dioxin), triggering the death of tenths of thousands of farm animals, either by the emanations or by slaughtering, while 193 children suffered from chloracne induced by the chemical. Several papers indicated that dioxin influences the development of the nervous system, as recently reviewed [129]. As an interface to the fetal organs, the placenta is particularly interesting to understand how fetal organs might be touched by the molecule. Recently, the mechanism of action of this molecule in the placenta has been elegantly analyzed mechanistically in a rat model comparing wild type and *Ahr-/-* animals [130]. TCDD exposure revealed placental adaptation at low to medium doses, up to a certain level of contamination, leading eventually to pregnancy termination. The authors showed that the aryl hydrocarbon receptor (AHR), a major factor of the detoxication cascades, as well as its major xenobiotic managing pathway target, CYP1A1, were activated at the implantation site in the endothelial cells at the maternal uterine side (not on the placenta itself). The authors concluded their study by an interesting single-cell RNA seq analysis showing cell-specific alterations in various cell populations (natural killer cells, macrophages, endothelial cells) at the uterine placental interface.

### 3.5. Exposure to Organometallic Molecules and Endocrine Disrupters

Tributyltin (TBT) is a persistent organotin pollutant widely used as agricultural and wood biocides for more than 40 years. Studies in mice have shown that it adversely impacts pregnancy by inducing developmental disorders of the placenta, including dysregulation of key molecules, impairing proliferation, inducing apoptosis, and oxidative stress. TBT administration increased levels of MDA and H_2_O_2_ and decreased activities of catalase and SOD [131]. Tributyltin also plays the role of an endocrine disrupter, as other organotins are supposed to act. A study in rats showed that organotin-contaminated sea food triggers abnormal histology of the placenta, accompanied by an excess of OS [132]. The effects of endocrine disrupters may be equivocal, as one study on the rat model indicates that exposure to genistein (a natural estrogeno-mimetic abundant in soy) modifies the OS status of the placenta [133]. Rats force-fed with genistein presented an increase of antioxidant levels (SOD, GSH, CAT) at gestational days 18 and 20 in the circulating blood, while an opposite effect was apparent in the placenta and amniotic fluid. According to the authors, this profile marks a positive effect of genistein attenuating OS in the placenta.

### 3.6. Plastic Modifiers: Phenols, Bisphenols and Parabens

Synthetic phenols constitute a family of chemicals supposed to have endocrine disruption properties. They are widely used to produce polycarbonate and epoxy resins, as well as ultraviolet filters, biocides (such as insecticides), antimicrobial agents for the fabrication of personal care products and plastics, and have become ubiquitous environmental contaminants. The general population is widely exposed to these molecules which are readily detected in placental tissues [105,134,135,136]. Studies in humans and animal models suggest that exposure to these compounds may be related to several adverse health outcomes, including pregnancy complications [137,138,139,140,141,142].

Imprinted genes are known to be targeted by these molecules [143], suggesting possible genomic (epigenetic effects through methylation alteration, as recently explored for nine molecules (bisphenolA -BPA-, benzophenone3, triclosan, 2.4 and 2.5 dichlorophenol, butyl- ethyl, methyl, and propylparaben). Altered methylation in the placentas was observed for 46 DMRs following exposition to triclosan (37 DMRs), confirming alterations nearby imprinted genes, nevertheless without specific alterations nearby genes directly involved in OS metabolism [144]. Consistently, Basak and coworkers showed in the trophoblast model HTR8/SV-neo that BPA exposure (1 nM) decreased promoter methylation of genes involved in metabolic and OS, such as GSR, PRDX2, GPX7, and GPX3, among others [145].

Examination of the mode of action of phthalates and parabens in the liver has also shown that they induce OS through the inhibition of the enzymatic activities of SOD, CAT, and GPX [146,147].

Tri-ortho-cresyl phosphate (TOCP), used as plasticizers, plastic softener, and flame-retardant, was reported to cause reproductive toxicity in mammals. In a recent study, dams were orally administered different doses of TOCP to explore its effects on placental development [148]. TOCP exposure significantly reduced numbers of the implanted embryo and adversely affected placental anthropometry. In addition to inducing apoptosis and autophagy, TOCP exposure increased the production of H_2_O_2_ and MDA. The induction of OS could be explained by an observed marked reduction in the activities of the catalase and SOD enzymes.

On the JEG-3 trophoblast cell model, the toxicity of BPA was evaluated at an E50 of 138–219 µM, while phthalates did not display apparent cytotoxicity [149]. In the trophoblast cell model BeWo placed in harsh conditions promoting OS, exposure to BPA has been shown to decrease ROS production while activating the expression of BCL2 (an antiapoptotic protein), increasing glutathione levels, decreasing apoptosis markers, and thus seemingly protecting the cells against cell death [150]. Mechanistically, BPA was able to limit the antioxidant response through limiting the activation of genes encompassing the ARE in their promoters, and this through impairing the expression and nuclear translocation of Nrf2 (a transcription factor binding to the ARE, paramount for managing the antioxidant response in various cell types, [89]).

Studies in humans and animal models suggest that exposure to these compounds may be related to several adverse health outcomes, including pregnancy complications [137,138,140,141,142,151]. A recent paper used the sheep model to test placental function following exposure to BPA by subcutaneous injection, days 30 to 90 post-fertilization, 0.5 mg/kg [152], showing that at gestational day 65, the fetal weight and the placental efficiency are reduced. The authors found an increase in lipid peroxidation, and antioxidant levels (GSR mRNA, SOD1 mRNA, SOD2 mRNA), with an increased nitrosylation of protein and dityrosine production. These observations suggest that oxidative and nitrosative stress are activated and that the antioxidant machinery is activated in the placenta following BPA exposure.

## 4. Final Considerations and Conclusions

In this review, we attempted to connect three concepts: (1) environmental exposure to contaminants, (2) the induction of oxidative stress in the placenta and (3) obstetric diseases of placental origin. The mechanistic studies that we present seem to demonstrate that the impact of environmental pollutants upon the health risk is genuine and that the generation of oxidative stress in the placenta is a sensible and truthful mediator of placental diseases. As such, in the STOX1 mouse model of preeclampsia, we have shown previously that oxidative/nitrosative stress is a major cause of the onset of the disease [153,154], corroborating the importance of this pathway in hypertensive disorders of pregnancy [155,156].

Specific environmental pollutants are now produced *en masse* for novel technological developments, for instance, connected to the upsurge of electric vehicles in many countries, leading to an exponential production of batteries that may release novel molecules in our environment. These batteries depend upon the use of rare elements (neodymium, lanthanum, terbium, dysprosium, lithium, cobalt that may reveal their effect on placental health in the future). For example, an ancient German publication revealed that in highly contaminated environments, traces of lanthanum and bromide are detectable in the placentas [157]. There is more recent literature regarding lithium exposure, showing, for instance, in an Argentinian mother-child cohort, that elevated exposure to this metal as well as to boron, arsenic, and antimony induced a reduction of the telomere length in the placenta (especially through arsenic exposure, while lithium apparently induced elongation of the telomeres in maternal tissues [158]). Earlier, lithium in the maternal blood was shown negatively associated with all fetal measures of size [159]. By itself, lithium has been recurrently associated with oxidative stress, including in placental cells [160]. Cobalt is also a potential risk factor since exposure to this metal in cells mimics hypoxia, by itself a potential cause of oxidative stress. Besides these technological novelties in transportation, other industrial developments linked to ecological concerns, such as recycling activities (for instance, of electronic material, called ‘e-waste’), increase the risk of propagating novel molecules in the atmosphere. Recently, a meta-analysis revealed that amongst 20 studies [161], one explored DNA damage in the placenta [162] and found reduced telomere length in this organ; in this study, cadmium placental concentrations were associated with the phenotype; since oxidative stress (such as caused by cadmium exposure) is linked to telomer attrition [163], this is a possible mechanism, albeit not directly studied in the paper. The authors surmised that improper e-waste processing was the primary cause of the exposure. In the future, such exposition may gain in importance. Other important sources that can be expected are flame retardants such as polybrominated biphenyl esters (PBDE, [164,165]), polychlorobiphenyls (PCBs), or other molecules. Overall, recycling consequences of e-waste exposure in China have been reviewed, including in the placenta [166]. Plausible health consequences remain an important preoccupation for the future.

**Figure 3:**
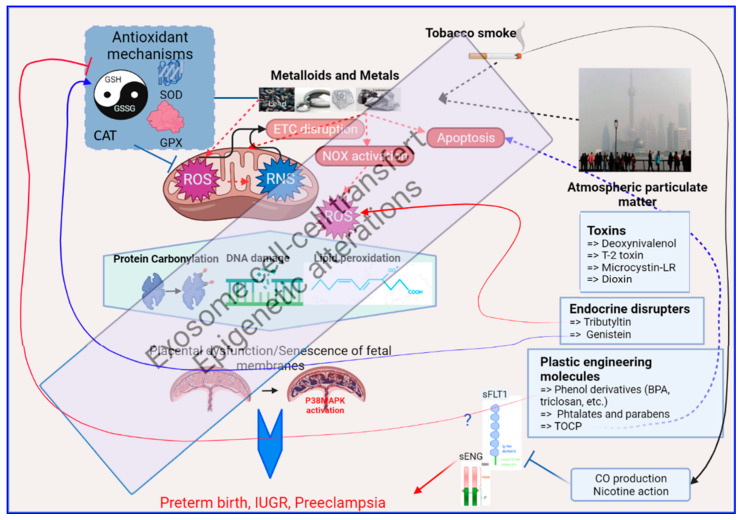
A brief graphic summary of the mechanistic impacts of environmental exposure through oxidative stress alterations in the human placenta, as detailed in the text of this review. At the center of this graph is the mitochondria which is the major producer of oxidative stress (ROS = Reactive Oxygen Species, and RNS = Reactive Nitrogen Species). The red dashed arrows relate to the generation of oxidative stress; the box on the upper left summarizes the antioxidant mechanisms that will fight against oxidative stress (the balance GSH/GSSG, the Superoxide Dismutases, the Glutathione peroxidases, the Catalase). The effect of OS on biomolecules appears on proteins, DNA and lipids as shown in the hexagon in the middle of the figure, with specific chemical modifications. Below, the placenta on which this review is focused is presented, part of the oxidative stress response being triggered in this organ by the activation of the P38/MAPK pathway. In black are the sources of oxidative stress in the human placenta that are described in this paper (Metalloids and Metals, Tobacco smoke and Atmospheric particulate matter). In Blue boxes at the right part of the figure are presented specific chemicals that are part of the placental regulation of oxidative stress, acting either on ROS production or modulation of ROS detoxification that is further detailed in the text. Other abbreviations: ETC = Electron Transport Chain, NOX = Nitric Oxidases, sFLT1 = Soluble FMS-Like Tyrosine kinase 1, sENG = soluble Endoglin, CO = Carbon Monoxide, TOCP = Tri-ortho-cresyl phosphate.

In conclusion, the induction of oxidative stress in the placenta by environmental toxicants has been studied in a very limited number of cases (as summarized in Figure 3). Similar to other tissues or organs, these studies point to the mitochondria and the cellular antioxidant activity (both enzymatic and non-enzymatic) as main targets. However, to design effective therapeutic approaches, more studies using in vivo and in vitro models are required to investigate the precise mechanisms involved in the induction of oxidative stress by at least the principal pollutants known to impact pregnancy.
